# Quantitative static contrast-enhanced phantom study to establish performance of advanced cone beam computed tomography for functional imaging applications

**DOI:** 10.1016/j.phro.2026.101125

**Published:** 2026-11-07

**Authors:** Riley McMaster, Raanan Marants, Hedi Mohseni, Catherine Coolens

**Affiliations:** a Department of Medical Biophysics, University of Toronto, Toronto, Ontario, Canada; b Princess Margaret Cancer Centre, University Health Network, Toronto, Ontario, Canada; c Department of Radiation Oncology, University of Toronto, Toronto, Ontario, Canada

**Keywords:** advanced cone beam computed tomography, quantitative image quality, contrast-enhanced phantoms, dynamic contrast-enhanced imaging, functional measurements

## Abstract

**Background and purpose:**

Dynamic contrast-enhanced computed tomography (DCE-CT) is a quantitative imaging technique that measures vascular perfusion. Extending this to cone beam CT (CBCT) would enable functional measurements on radiotherapy machines. An advanced CBCT imaging solution, with enhanced image quality and rapid acquisitions, offers potential. However, the static quantitative and spatial performance of advanced CBCT relevant to DCE imaging has not been characterized.

**Materials and methods:**

To address this, the image quality of advanced CBCT was compared with standard CBCT and CT using contrast-enhanced phantoms with DCE-specific characteristics not previously applied to CBCT. A contrast enhancement CT (CECT) phantom (acrylic cylinder with capsule inserts) and a humanoid phantom (human torso with a liver containing tumour-mimicking spheres) were imaged across different acquisition settings.

**Results:**

Using the CECT phantom, advanced CBCT demonstrated improved CT number accuracy compared to standard CBCT (<23.9 vs. <78.9 HU) and greater CT number constancy (<8.4 vs. <12.9 HU). Iterative reconstruction on advanced CBCT offered improvements over filtered back-projection. Using the humanoid phantom, findings were confirmed with CT number accuracy (<7.1 vs. <42.8 HU), CT number variation (<11.3 vs. <49.8 HU) and volume accuracy (<128.8 vs. <1067.3 mm^3^). Advanced CBCT achieved spatial resolutions of 2.3–5.0 lp/cm across different acquisitions and measurements, meeting the 5 lp/cm clinical benchmark under certain conditions. It also displayed stable CT number–density calibration.

**Conclusions:**

Advanced CBCT compared to standard CBCT improved image quality, approaching CT-level performance. Findings demonstrated robust volume and contrast-specific quantification under DCE conditions, supporting further evaluation of functional CBCT.

## Highlights

• Evaluated advanced cone beam computed tomography with contrast-enhanced phantoms.
• Advanced cone beam computed tomography improved accuracy in contrast and volume.
• Advanced cone beam computed tomography achieved up to 5 lp/cm spatial resolution.
• Advanced cone beam computed tomography showed stable CT number–density calibration.

## Graphical abstract

**Figure:**
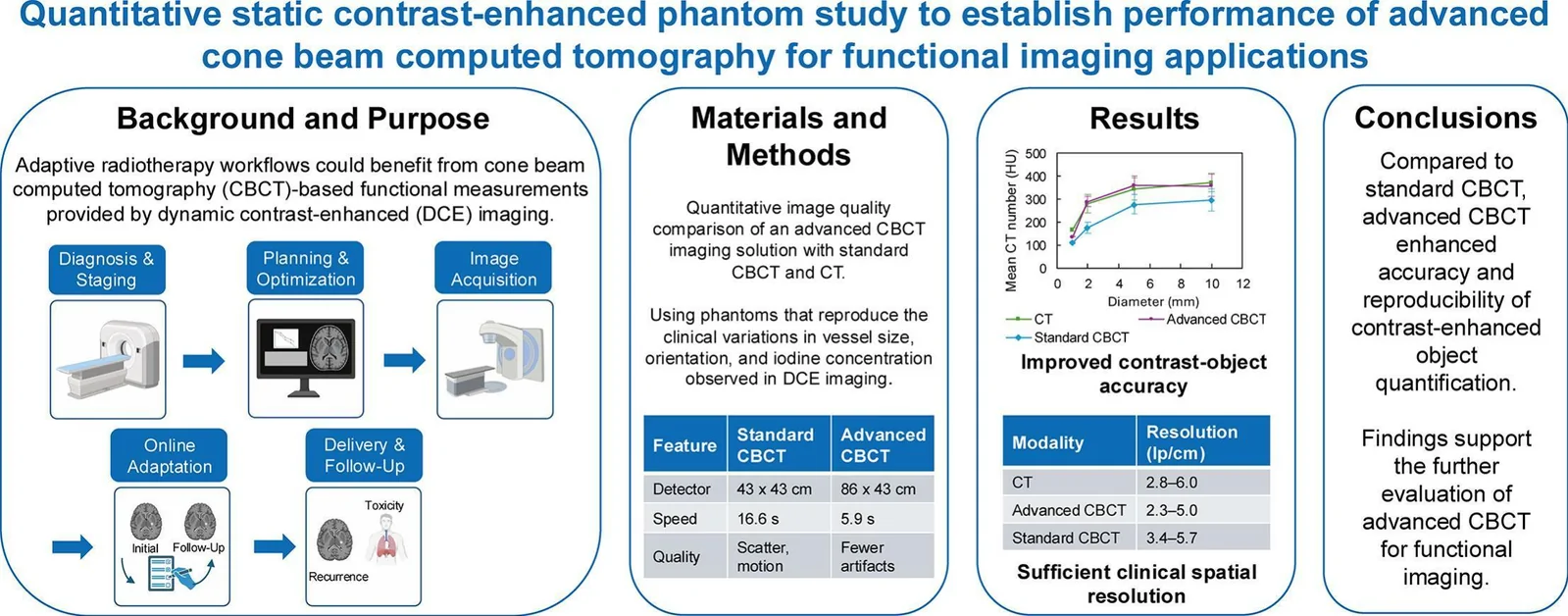

## 1 Introduction

Approximately 50% of cancer patients require radiotherapy, emphasizing the need to optimize treatment workflows [1], [2]. Image-guided radiation therapy (IGRT) has improved treatment accuracy by incorporating imaging directly into radiotherapy systems [3], [4]. While cone beam computed tomography (CBCT) is primarily used for anatomical guidance in IGRT, CT-based imaging can also provide quantitative functional information [3], [4], [5], [6]. Dynamic contrast-enhanced CT (DCE-CT), for example, measures tumour vascular perfusion following intravenous contrast administration [6], [7], [8]. These hemodynamic measurements support diagnosis, staging, and early assessment of treatment response [6], [7], [8].

Integrating functional measurements into radiotherapy workflows could improve target localization, enable personalized treatment, reduce repeat CT scans, and consolidate imaging and treatment [8], [9]. However, CBCT's long acquisitions and limited image quality increase susceptibility to patient motion and photon scatter, reducing the signal-to-noise ratio (SNR) and limiting kinetic analysis [5], [6], [7], [8], [9]. An advanced CBCT imaging solution offering enhanced image quality and faster scanning may overcome these limitations [10].

Previous phantom studies of advanced CBCT have demonstrated reduced metal artifacts and motion blur, improved CT number accuracy and SNR, enhanced target volume delineation, extended field-of-view (FOV) reconstruction, and characterized spatial resolution [10], [11], [12], [13], [14], [15]. These studies primarily evaluated image quality to determine whether advanced CBCT could support radiotherapy applications traditionally requiring simulation CT. Subsequent studies compared dose calculations and treatment plans derived from advanced CBCT and CT, supporting simulation-free adaptive radiotherapy [16], [17], [18], [19], [20]. Studies of contrast-enhanced CBCT have also demonstrated improved tumour visualization and detection [17], [21], [22].

Although previous studies have established the image quality, accuracy, and spatial resolution of advanced CBCT and demonstrated the benefits of contrast-enhanced CBCT, they have not specifically evaluated image characteristics relevant to future DCE applications [10], [11], [12], [13], [14], [15], [16], [17], [18], [19], [20]. Accurate and reproducible spatiotemporal representation of contrast enhancement is needed to characterize arterial input functions (AIFs), tissue time-attenuation curves (TACs), and subsequent perfusion parameters [6], [7], [8]. This requires reliable visualization and delineation of contrast-enhancing vessels and lesions across varying sizes, orientations, and contrast concentrations. Measurements rely on accurate representation of structures at individual time points. Therefore, static characterization of contrast detection and spatial fidelity provides a controlled first step before evaluating dynamic contrast behaviour.

Accordingly, this study compared advanced CBCT, standard CBCT, and CT using contrast-enhanced phantoms designed to evaluate DCE-relevant characteristics and not previously applied to CBCT imaging. This enabled assessment of contrast representation, volume definition, spatial resolution, and CT number–density calibration for subsequent functional imaging applications. Advanced CBCT was hypothesized to improve quantitative performance over standard CBCT, approaching that of CT.

## 2 Materials and methods

### 2.1 Advanced CBCT description

The advanced CBCT (HyperSight, Varian Medical Systems) is available for the Halcyon O-ring and TrueBeam C-arm linear accelerators (LINACs). Both systems feature a 43 × 86 cm detector panel, a thicker cesium iodide scintillator providing twice the dose efficiency of previous generations, and a reduced grid-to-scintillator distance minimizing photon loss [10]. In addition to conventional Feldkamp-Davis-Kress (FDK) filtered back-projection reconstruction, the system offers iterative CBCT reconstruction (iCBCT), iCBCT with Acuros scatter correction (Acuros), and iCBCT with metal artifact reduction (MAR) [23]. On the Halcyon, a maximum gantry rotation speed of 36°/s enables scans to be completed in 5.9 s.

### 2.2 Imaging protocols

Advanced CBCT scans were acquired on the Halcyon and TrueBeam and compared with standard CBCT scans acquired on a separate TrueBeam. A CT simulation scanner (Acquilion One, Canon Medical Systems) provided benchmark data. Acquisition parameters were matched where possible, subject to CBCT system constraints. Protocols were selected to represent highly vascular tumours for which DCE imaging may be beneficial, including those of the lungs, liver, and pancreas [24].

CT scans were acquired using an abdominal protocol in helical and volume mode. CBCT scans were acquired using the half- or full-fan configuration. Half-fan acquisitions use an offset X-ray panel and a 360° gantry rotation to provide an extended FOV, whereas full-fan acquisitions use a centered panel and a 211° rotation, resulting in a smaller FOV. Both TrueBeam systems used thorax (half-fan) and liver (full-fan) protocols with a gantry rotation speed of 9°/s. Halcyon scans used a thorax protocol (half-fan; 6°/s) and one without a predefined organ-specific preset (full-fan; 36°/s). The latter configuration was selected for its rapid acquisition, which is applicable to future functional imaging.

Five test scenarios were evaluated: (1) default settings; (2) variable tube-current exposure (150–312 mAs); (3) variable tube voltage (80–140 kV); (4) variable phantom placement (40 mm left, right, anterior, posterior, superior, or inferior to isocenter); and (5) variable reconstruction algorithm on the Halcyon (FDK, iCBCT, iCBCT Acuros, and iCBCT MAR). Two replicate scans were acquired for each scenario to account for random fluctuations. Detailed acquisition parameters are provided in supplementary material A.

### 2.3 Imaging phantoms

The contrast enhancement CT (CECT) phantom was designed in-house for DCE quality assurance (QA) and protocol optimization and was imaged under all test scenarios. It simulates physiological concentrations of iodixanol contrast agent in tissues and vessels following intravenous administration (1.5–15 mgI/mL) [25]. The acrylic cylinder contains capsules made from polystyrene (1.05 g/cm^3^), ultem (1.27 g/cm^3^), delrin (1.41 g/cm^3^), and torlon (1.56 g/cm^3^), representing a range of CT numbers (Hounsfield units, HU). The capsules were 20 mm long and varied in diameter (1, 2, 5, and 10 mm) and orientation: axial capsules had their long axis aligned with the y-axis, transaxial capsules with the z-axis, and diagonal capsules with the x-y plane. The phantom design supports DCE analysis, enabling volume and spatial definition for orientation-dependent vessel quantification.

The humanoid phantom was also designed in-house to reproduce physiological levels of contrast enhancement in metastases and was imaged only under default settings [26]. This anatomically accurate human torso model was constructed from Rando® material (with CT numbers of 20–30 HU at 120 kV). It contained liver inserts of varying diameter (1.6, 3.2, 4.8, 6.4, 7.9, 9.5, and 12.7 mm), composition (acrylic or custom polyurethane with antimony to produce CT numbers of 37–131 HU at 120 kV) and location (superior or inferior). Images of both phantoms are provided in supplementary material B.

### 2.4 Image analysis

A Python script was developed to automate object analysis in both phantoms. Object centroids were identified using a template, and three-dimensional regions of interest (ROIs) were separated from the background using Otsu thresholding [27]. Binary masks were generated for the 1.56-g/cm^3^ capsules in the CECT phantom at each orientation and diameter (12 masks) and for the acrylic spheres in the humanoid phantom at each diameter (seven masks), as these objects provided the highest SNR. The resulting masks were then applied to the remaining objects based on orientation and diameter in the CECT phantom and diameter in the humanoid phantom. Mean CT number, standard deviation (SD), and volume were calculated for each object, with measurements averaged across replicates.

### 2.5 Image quality assessment

To quantify image noise in the CECT phantom (1.56-g/cm^3^, 10-mm capsules), CT number constancy and volume constancy were calculated as the SD across dose levels (mAs) and positions within the phantom. CT number uniformity was calculated as the coefficient of variation at default settings (Eq. 1).

(1) $$\text{Uniformity}=\frac{\sigma }{\mu }∙100\%$$

σ: voxel-wise SD.

μ: mean across voxels.

CT number accuracy and volume accuracy were calculated as the absolute difference between measured and reference values at default settings, after averaging across capsule orientations in the CECT phantom and sphere locations in the humanoid phantom (Eq. 2). Reference CT numbers and volumes were obtained from previously published measurements of the same phantoms acquired using diagnostic CT at 120 kV [25], [26].

(2) $$\text{Accuracy}=\left|\mu -X_{\mathrm{ref}}\right|$$

μ: mean across voxels.

$X_{\mathrm{ref}}$: reference value.

CT number variation and volume variation were also calculated as the SD across capsule orientations in the CECT phantom and sphere locations in the humanoid phantom.

Spatial resolution was evaluated using the CECT phantom (1.56-g/cm^3^, 10-mm capsules) at default settings and with variable reconstruction. An algorithm by Thornton et al. was adapted to compute modulation transfer function (MTF) curves [28]. For each replicate, the object centroid was determined, and the surface-spread and plane-spread functions were calculated and Fourier-transformed to obtain the MTF curves. Curves were averaged for each direction, and the spatial frequency at the 10% threshold was reported in line pairs per centimeter (lp/cm). Additional details are provided in supplementary material C.

CT number–density calibration was evaluated using the CECT phantom at different tube voltages. This relationship is important for tissue inhomogeneity correction, dose calculation, machine-specific standardization, and pharmacokinetic modelling in quantitative imaging [8], [29], [30]. Additional details are provided in supplementary material D.

## 3 Results

For the CECT phantom, CT scans exhibited CT number constancy below 2.0 HU and volume constancy below 25.8 mm^3^. CT number accuracy and volume accuracy were below 9.1 HU and 24.4 mm^3^, respectively (Table 1). For full-fan acquisitions, advanced CBCT on the Halcyon compared to standard CBCT improved CT number constancy (<6.5 vs. <12.9 HU), CT number accuracy (11.1 vs. 78.9 HU) and CT number uniformity (<13.8 vs. <14.3%). Iterative reconstruction further reduced CT number accuracy to 0.9 HU with Acuros, and 0.6 HU with MAR. Differences between half- and full-fan acquisitions were minimal, except for standard CBCT with CT number accuracies of 4.4 HU and 78.9 HU, respectively.

**Table 1:** Image quality metrics for the CECT phantom (1.56-g/cm^3^, 10-mm capsules). CT number constancy and volume constancy were reported as the range across capsule orientations (axial, transaxial, and diagonal). CT number and volume were averaged across orientations prior to accuracy calculations.

Imaging modality	Scan mode / configuration (reconstruction algorithm)	CT number constancy across mAs (HU)	CT number constancy across phantom placement (HU)	Volume constancy across mAs (mm^3^)	Volume constancy across phantom placement (mm^3^)	CT number uniformity (%)	CT number accuracy (HU)	Volume accuracy (mm^3^)
CT	Helical	0.3–0.7	1.3–2.0	1.3–5.4	7.6–25.8	10.6–10.9	1.2	24.4
	Volume	0.1–0.3	0.9–1.7	1.4–2.4	1.9–6.1	10.1–10.3	9.1	6.7
Advanced CBCT (Halcyon)	Half-fan (FDK)	1.0–1.5	5.1–7.9	5.8–9.9	13.7–80.5	12.9–13.4	11.2	2.0
	Full-fan (FDK)	–	5.5–6.5	–	17.7–67.2	12.8–13.8	11.1	35.4
	Full-fan (iCBCT)	–	–	–	–	–	2.7	30.3
	Full-fan (iCBCT Acuros)	–	–	–	–	–	0.9	24.4
	Full-fan (iCBCT MAR)	–	–	–	–	–	0.6	25.1
Advanced CBCT (TrueBeam)	Half-fan (FDK)	0.3–0.7	6.2–8.4	6.4–16.5	13.9–27.3	11.9–14.2	17.7	28.3
	Full-fan (FDK)	0.3–0.5	2.5–6.8	4.0–7.6	5.6–21.8	11.9–13.1	23.9	21.3
Standard CBCT (TrueBeam)	Half-fan (FDK)	1.1–1.6	8.1–10.5	5.9–12.9	11.0–55.6	12.4–12.7	4.4	13.0
	Full-fan (FDK)	0.3–0.7	8.6–12.9	3.4–5.0	10.9–40.7	13.8–14.3	78.9	25.5

CT number decreased with decreasing capsule diameter (Fig. 1a). Compared with advanced CBCT, standard CBCT showed greater volume deviation from CT for the 2- and 5-mm capsules (Fig. 1b-c). Across capsule orientations, CT exhibited CT number variation of 0.9 HU and volume variation of 6.1 mm^3^. For advanced CBCT on the Halcyon, the corresponding variations were 12.6 HU and 17.6 mm^3^, comparable to those observed on both TrueBeam systems. On the Halcyon, iterative reconstruction reduced CT number variation from 12.6 HU with FDK to 2.9 HU with Acuros and 2.8 HU with MAR, while volume variation remained comparable across algorithms.

**Fig. 1:**
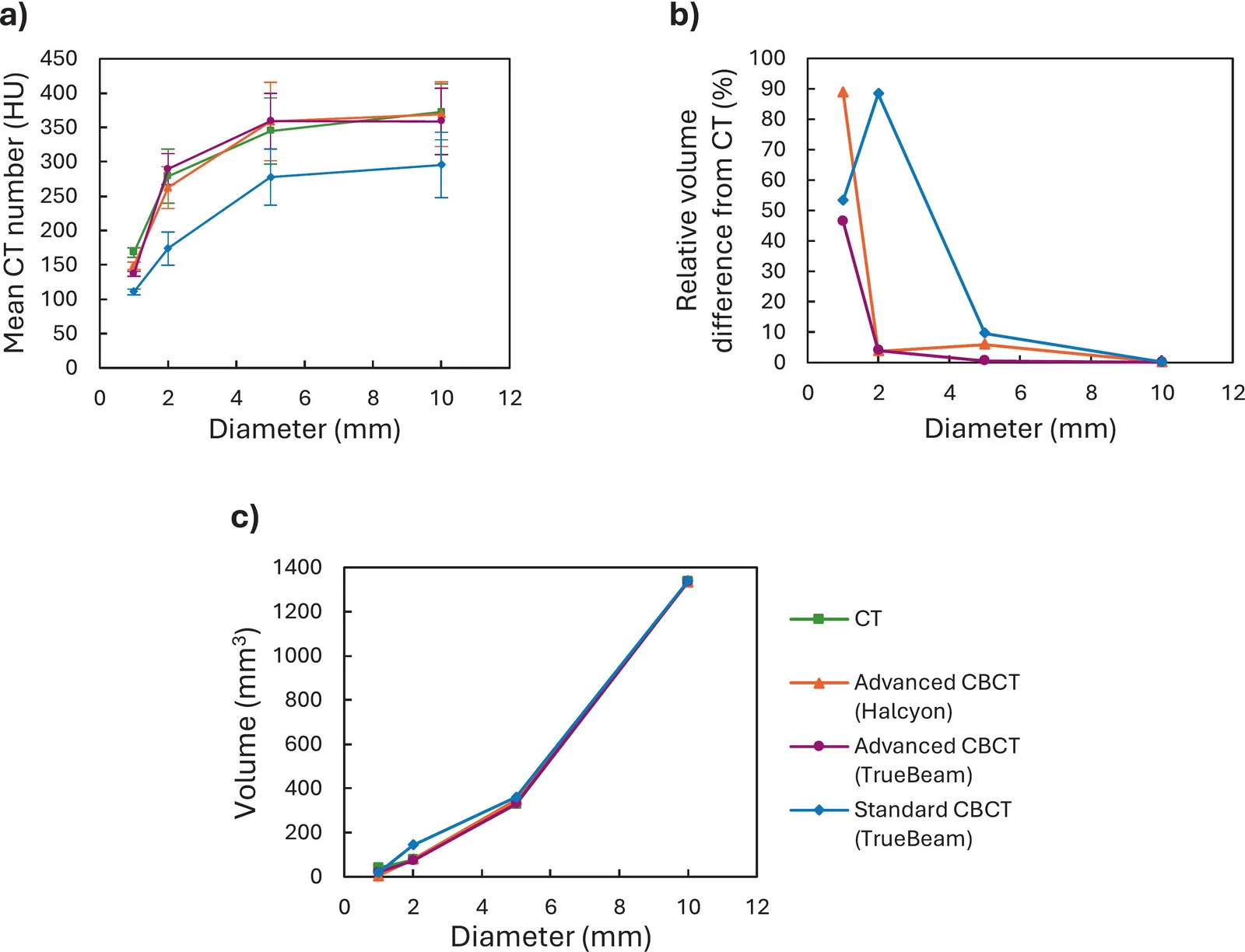
Variation in a) mean CT number; b) relative volume difference from CT; and c) absolute volume as a function of diameter for the axial, 1.56 g/cm3 capsules from the contrast enhancement CT (CECT) phantom. Imaged at 120 kV for CT and 125 kV for CBCT. CT reconstructed with iterative algorithm AIDR3 in volume mode; advanced CBCT reconstructed with iterative CBCT Acuros in a full-fan configuration; and standard CBCT reconstructed using the FDK filtered back-projection algorithm in a full-fan configuration. Error bars represent within-capsule standard deviation of voxel-wise CT number.

For the humanoid phantom, advanced CBCT on the Halcyon in the full-fan configuration exhibited improvements in CT number accuracy compared with standard CBCT (2.4 vs. 42.8 HU), as well as in CT number variation (10.1 vs. 49.8 HU) and volume accuracy (11.8 vs. 1067.3 mm^3^) (Table 2).

**Table 2:** Image quality metrics for the humanoid phantom (acrylic, 12.7-mm spheres). CT number and volume were averaged across sphere locations (superior and inferior) prior to accuracy calculations.

Imaging modality	Scan mode / configuration	CT number accuracy (HU)	Volume accuracy (mm^3^)	CT number variation across locations (HU)
CT	Helical	8.2	89.5	6.1
	Volume	9.9	145.2	9.4
Advanced CBCT (Halcyon)	Half-fan	1.1	128.8	11.3
	Full-fan	2.4	11.8	10.1
Advanced CBCT (TrueBeam)	Half-fan	0.2	87.1	10.8
	Full-fan	7.1	106.5	0.4
Standard CBCT (TrueBeam)	Half-fan	0.1	637.5	10.9
	Full-fan	42.8	1067.3	49.8

CT exhibited spatial resolutions of 4.3–4.5 lp/cm (Table 3). For full-fan acquisitions, advanced CBCT achieved a resolution of 3.0 lp/cm on the Halcyon and 4.1 lp/cm on the TrueBeam, compared with 3.8 lp/cm for standard CBCT. On the Halcyon, iterative reconstruction increased resolution to 3.6 lp/cm. On the TrueBeam, full-fan acquisitions yielded higher resolution than half-fan acquisitions for both advanced CBCT (4.1 vs. 3.6 lp/cm) and standard CBCT (3.8 vs. 3.4 lp/cm). Resolution on the Halcyon was comparable between half- and full-fan acquisitions. Across capsule orientations and MTF directions, resolution was lowest for the diagonal orientation and highest along the y-axis (Tables S2 and S3).

**Table 3:** 10% MTF values for the CECT phantom (1.56-g/cm^3^, 10-mm capsules). Measurements were reported with the median (range) across capsule orientations (axial, transaxial, and diagonal) and MTF directions (X, Y, and Z).

Imaging modality	Scan mode / configuration (reconstruction algorithm)	10% MTF (lp/cm)	Pixel spacing (mm)
CT	Helical	4.5 (3.2–5.7)	0.35
	Volume	4.3 (2.8–6.0)	0.35
Advanced CBCT (Halcyon)	Half-fan (FDK)	3.0 (2.3–4.8)	1.05
	Full-fan (FDK)	3.0 (2.3–5.0)	1.05
	Full-fan (iCBCT)	3.6 (2.3–4.6)	1.05
	Full-fan (iCBCT Acuros)	3.6 (2.3–5.0)	1.05
	Full-fan (iCBCT MAR)	3.6 (2.3–4.9)	1.05
Advanced CBCT (TrueBeam)	Half-fan (FDK)	3.6 (2.3–4.8)	0.95
	Full-fan (FDK)	4.1 (2.3–4.7)	0.55
Standard (TrueBeam)	Half-fan (FDK)	3.4 (1.8–4.5)	0.91
	Full-fan (FDK)	3.8 (2.3–5.7)	0.51

CT and advanced CBCT exhibited limited variation in CT number across tube voltages (Fig. 2). For standard CBCT, the separation between CT number–density curves increased with density, with CT number decreasing at higher tube voltages. On TrueBeam systems, tube voltage and scan configuration were partially confounded, as 80–100 kV acquisitions used half-fan, whereas 125–140 kV acquisitions used full-fan. Advanced CBCT on the Halcyon also exhibited limited variation in CT number across tube voltages when acquisitions were performed in both scan configurations (Fig. S5). Calibration procedures are summarized in Table S4.

**Fig. 2:**
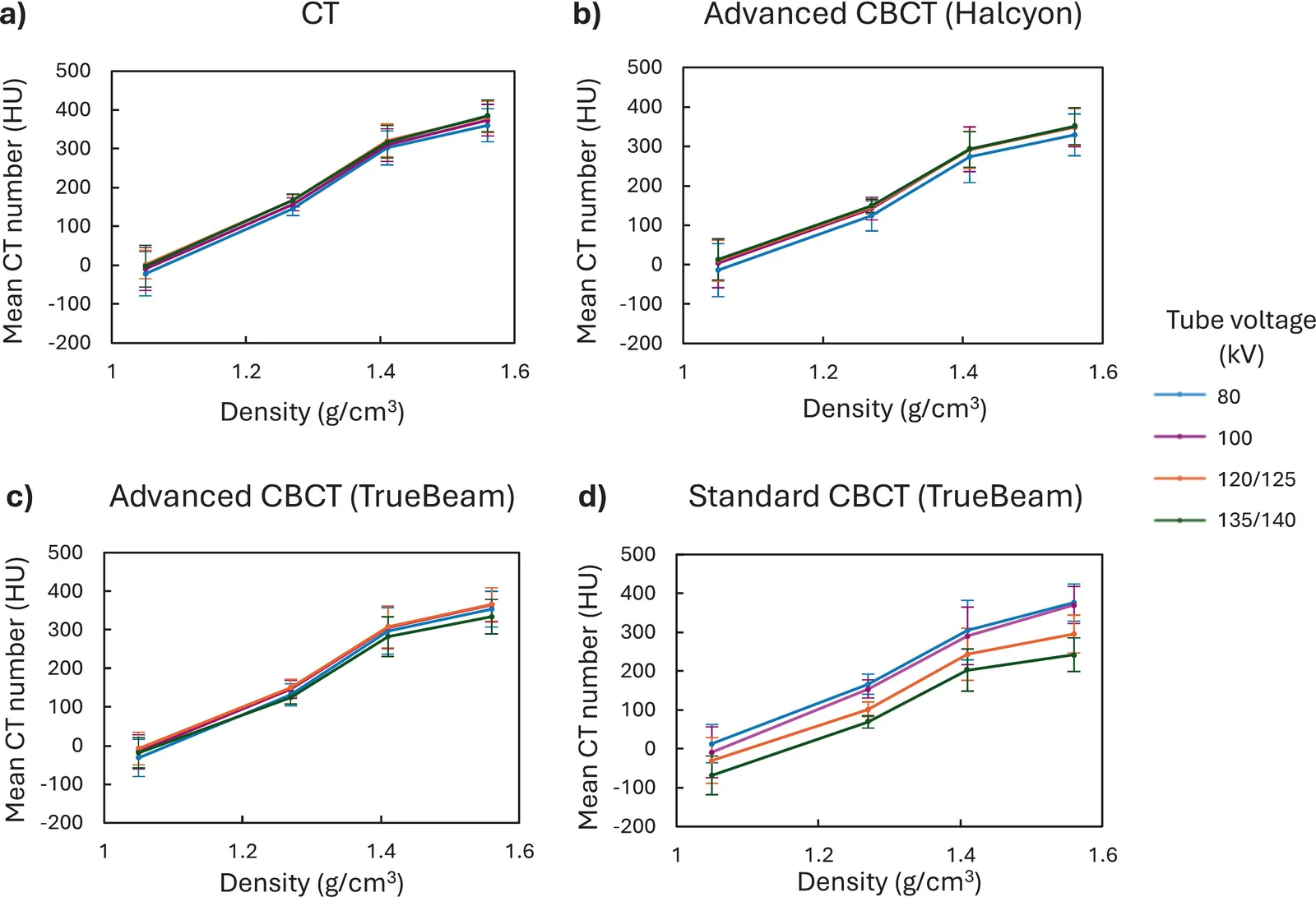
Variation in mean CT number as a function of density for the axial, 10 mm capsules from the contrast enhancement CT (CECT) phantom on a) CT; b) advanced CBCT (Halcyon); c) advanced CBCT (TrueBeam); and d) standard CBCT (TrueBeam) for different tube voltages. CT was evaluated at 80, 100, 120 and 135 kV, while CBCT was evaluated at 80, 100, 125 and 140 kV. CT reconstructed with iterative algorithm AIDR3 in volume mode; CBCT reconstructed using the FDK filtered back-projection algorithm. Advanced CBCT (Halcyon) imaged in a full fan configuration, while advanced CBCT (TrueBeam) and standard CBCT (TrueBeam) imaged in a half-fan configuration for 80–100 kV, and a full-fan configuration for 125–140 kV. Error bars represent within-capsule standard deviation of voxel-wise CT number.

## 4 Discussion

The larger CT number deviations observed with standard CBCT (Table 1, Table 2, and Fig. 1) likely reflect greater cupping, shading and scatter artifacts, which can reduce measured attenuation. These effects may be particularly pronounced in full-fan acquisitions due to increased projection data [5], [10], [23]. Advanced CBCT's greater detector efficiency, reduced photon loss and enhanced scatter correction likely reduced these effects [10], [23]. The improved CT number accuracy with iterative reconstruction was consistent with its forward-projection model, which is less susceptible to noise and artifacts than filtered back-projection [23], [31]. Larger voxels may also have affected CT number constancy and uniformity through signal averaging, alongside differences in noise properties, hardware and reconstruction performance. Additionally, CBCT dose levels (mAs) were approximated rather than matched to those of CT, which may have affected noise-dependent metrics.

These findings are consistent with reported CT number differences of up to 28 HU for Acuros-reconstructed advanced CBCT and 284 HU for FDK-reconstructed standard CBCT [32]. CT number accuracy was also comparable to studies reporting errors of 1% for Acuros-reconstructed advanced CBCT and 0.7% for CT [14], [33]. Differences in tube voltage between CT and CBCT (120 vs. 125 kV) may have contributed to discrepancies in CT number accuracy for high-density materials, alongside differences in beam filtration and X-ray tube design [31].

The decrease in CT number with decreasing capsule diameter is consistent with partial-volume averaging, whereby the measured attenuation signal likely included contributions from the surrounding acrylic material (Fig. 1a). The larger volume deviations observed with standard CBCT for smaller capsule diameters further highlight differences in object-size-dependent quantification among systems. Findings agree with reports of reduced CT number variation across different phantom sizes with advanced CBCT compared with standard CBCT [11].

The lower resolution of advanced CBCT on the Halcyon compared with the TrueBeam is consistent with the larger pixels of the Halcyon (Table 3). The lower resolution of standard CBCT compared with advanced CBCT on the TrueBeam, despite its smaller pixels, suggests additional detector contributions. The improvement in resolution with iterative reconstruction of the Halcyon, despite unchanged pixel spacing, further suggests reconstruction contributions.

Although standard CT scanners can achieve 6–8 lp/cm at 10% MTF for perfusion protocols, a resolution of 5 lp/cm has been suggested as the minimum acceptable for radiotherapy imaging. A maximum voxel volume of 2 mm^3^ has also been proposed to prevent the loss of physiological perfusion details [34], [35], [36], [37]. In this study, resolution reached 5 lp/cm in some imaging configurations, capsule orientations, and MTF directions. Results agree with previous reports of 3.5–5.0 lp/cm at 10% MTF for advanced CBCT and standard CBCT in full-fan configurations [14], [38].

The energy dependence of material attenuation may explain the decrease in CT number with increasing tube voltage observed with standard CBCT (Fig. 2). For systems that acquire images for dose calculation (CT, Halcyon), QA includes multi-kV CT number calibration. CT number constancy and dose-of-the-day are performed on all systems to verify calibration stability (Table S4). Both TrueBeam systems underwent the same procedures, without kV-specific CT number calibration. Nevertheless, advanced CBCT on the TrueBeam and Halcyon demonstrated limited CT number variation across tube voltages (Fig. 2 and S5). This suggests that detector response and image-processing corrections may also contribute, in addition to calibration.

This study characterized DCE-relevant conditions using phantoms representing clinical variability in iodine concentrations, vessel and lesion dimensions, and orientations. However, respiratory motion, which may influence abdominal contrast quantification, was not evaluated. Voxel dimensions and scan configurations could not always be standardized across systems, potentially influencing SNR, contrast detectability, and spatial resolution. Consequently, the relative contributions of voxel size, tube voltage, detector hardware and reconstruction could not be independently evaluated. Clinical implementation of functional CBCT will also require consideration of the safety and feasibility of repeated contrast administration.

Compared with standard CBCT, advanced CBCT enhanced accuracy and reproducibility of contrast-enhanced object quantification through improved CT number accuracy, volume definition and iterative reconstruction. Spatial resolution reached the clinical benchmark for some configurations and measurements. Together with stable CT number–density calibration, these findings characterize the quantitative and spatial performance of advanced CBCT under static contrast-enhanced conditions, supporting its further evaluation for functional CBCT.

## CRediT authorship contribution statement

**Riley McMaster:** Writing – review & editing, Writing – original draft, Validation, Methodology, Investigation, Formal analysis, Data curation, Conceptualization. **Raanan Marants:** Writing – review & editing, Conceptualization. **Hedi Mohseni:** Writing – review & editing, Conceptualization. **Catherine Coolens:** Writing – review & editing, Supervision, Resources, Funding acquisition, Conceptualization.

## Declaration of competing interest

The authors declare the following financial interests/personal relationships which may be considered as potential competing interests: This work was supported in part by Varian Medical Systems.
