# Supplementary material for: Quantitative static contrast-enhanced phantom study to establish performance of advanced cone beam computed tomography for functional imaging applications
Source: Phys Imaging Radiat Oncol. 2026 Nov 7;42:101125. doi: 10.1016/j.phro.2026.101125 (PMC13760967; doi:10.1016/j.phro.2026.101125)
Supplement: Supplementary file 1 [file mmc1.pdf]

Supplementary material

Quantitative static contrast-enhanced phantom study to establish performance of advanced cone beam computed tomography for functional imaging applications

Supplementary material A. Imaging scenarios

Table S1. Acquisition parameters for the five test scenarios. CT helical mode slice thickness was set to 2.0 mm to match the default slice thickness of CBCT, while the volume mode had a default slice thickness of 0.5 mm. Tube current-exposure was set to be as close as possible to 300 mAs (270-310 mAs) for CBCT, limited by CBCT user restrictions. AIDR3 is the default CT iterative reconstruction algorithm. Two replicate scans were acquired for each scenario to account for random fluctuations.

Test scenarios	Imaging modality	Scan mode / configuration	Tube current-exposure (mAs)	Tube voltage (kV)	Phantom placement	Reconstruction algorithm	Slice thickness (mm)	Acquisition time (s)
Default settings	CT	Volume	300	120	Isocenter	AIDR3	0.5	1
		Helical	300	120		AIDR3	2.0	1
	Advanced CBCT (Halcyon)	Half-fan	312	125		FDK	2.0	60
		Full-fan	272	125		FDK	2.0	6
	Advanced CBCT (TrueBeam)	Half-fan	301	125		FDK	2.0	40
		Full-fan	299	125		FDK	2.0	23
	Standard CBCT (TrueBeam)	Half-fan	286	125		FDK	2.0	40
		Full-fan	300	125		FDK	2.0	23
Variable tube current-exposure (mAs)	CT	Volume	150, 200, 250, 300	120	Isocenter	AIDR3	0.5	1
		Helical	150, 200, 250, 300	120		AIDR3	2.0	1
	Advanced CBCT (Halcyon)	Half-fan	152, 197, 250, 312	125		FDK	2.0	60
	Advanced CBCT (TrueBeam)	Half-fan	159, 195, 248, 301	125		FDK	2.0	40
		Full-fan	150, 200, 250, 299	125		FDK	2.0	23
	Standard CBCT (TrueBeam)	Half-fan	152, 197, 250, 286	125		FDK	2.0	40
		Full-fan	150, 200, 250, 300	125		FDK	2.0	23
Variable tube voltage (kV)	CT	Volume	300	80, 100, 120, 135	Isocenter	AIDR3	0.5	1
		Helical	300	80, 100, 120, 135		AIDR3	2.0	1
	Advanced CBCT (Halcyon)	Full-fan	272	80, 100, 125, 140		FDK	2.0	6
		Half-fan	301	80, 100		FDK	2.0	40

	Advanced CBCT (TrueBeam)	Full-fan	299	125, 140		FDK	2.0	23
	Standard CBCT (TrueBeam)	Half-fan	286	80, 100		FDK	2.0	40
		Full-fan	300	125, 140		FDK	2.0	23
Variable phantom placement	CT	Volume	300	120	40 mm left, right, anterior, posterior, superior, inferior of isocenter	AIDR3	0.5	1
		Helical	300	120		AIDR3	2.0	1
	Advanced CBCT (Halcyon)	Half-fan	312	125		FDK	2.0	60
		Full-fan	272	125		FDK	2.0	6
	Advanced CBCT (TrueBeam)	Half-fan	301	125		FDK	2.0	40
		Full-fan	299	125		FDK	2.0	23
	Standard CBCT (TrueBeam)	Half-fan	286	125		FDK	2.0	40
		Full-fan	300	125		FDK	2.0	23
Variable reconstruction algorithm	Advanced CBCT (Halcyon)	Full-fan	272	125	Isocenter	FDK, iCBCT, iCBCT Acuros, iCBCT MAR	2.0	6

For the test scenario with variable tube current-exposure (mAs), scans were only acquired in the half-fan configuration for the Halcyon as the full-fan configuration had a non-adjustable mAs setting. For the test scenario with variable tube voltage (kV), a constant scan configuration was maintained for the Halcyon with all acquisitions in full-fan, and for CT with all acquisitions in volume mode. However, for TrueBeam, this was not possible as the half-fan configuration was restricted to 80 and 100 kV, while the full-fan configuration was restricted to 125 and 140 kV; therefore, a combination of both configurations was used. Because of this limitation, this experimental setup was also performed for the Halcyon, imaging 80–100 kV in half-fan and 125–140 kV in full-fan, as a comparative reference, included in supplementary material D. An additional test scenario investigating variable reconstruction was included only for the Halcyon in the full-fan configuration to evaluate whether the advanced reconstruction algorithms could improve the image quality of the fast acquisition protocol (full-fan; 36 °/s).

Supplementary material B. Imaging phantoms

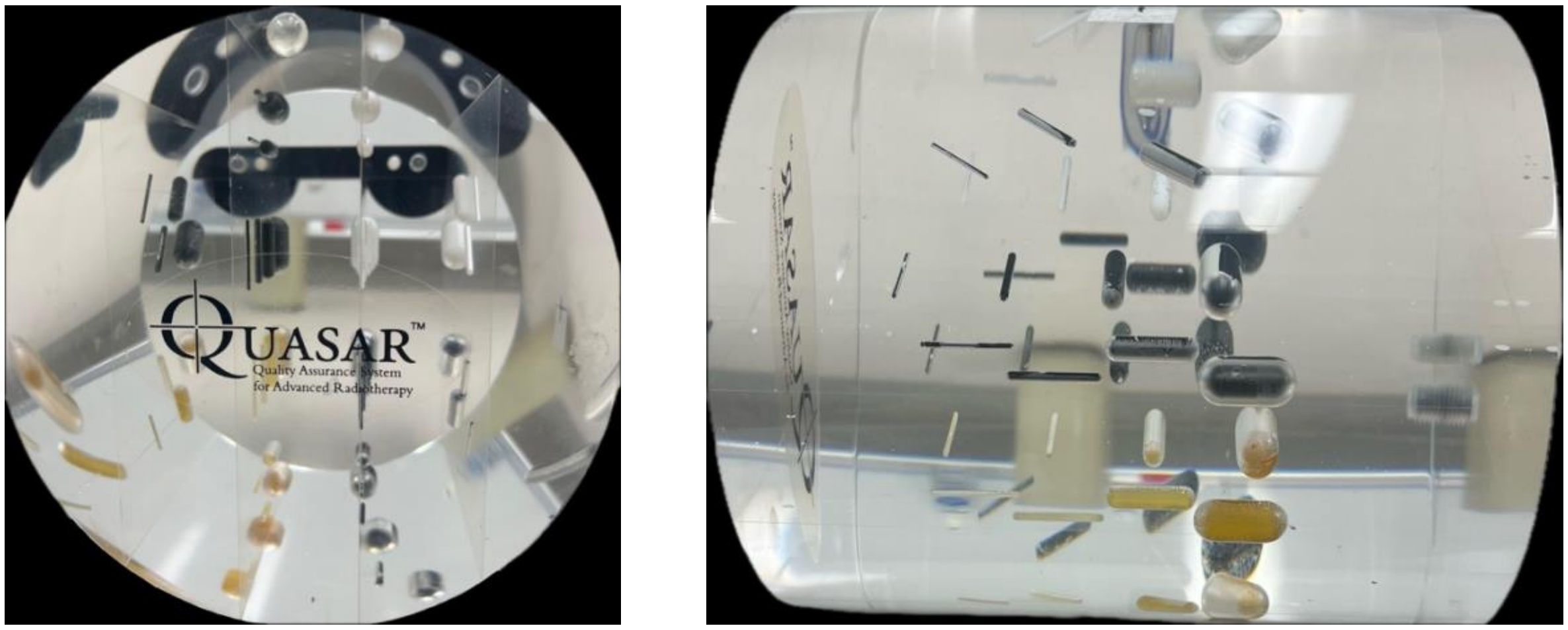

a) **b)**

Fig. S1. a) Front and b) side view of the contrast enhancement CT (CECT) phantom, designed in-house but is now commercially available [1]. An acrylic cylindrical phantom measuring 18 cm in length with a diameter of 16 cm, containing 48 capsules of varying diameter (1, 2, 5, and 10 mm), orientation (axial, transaxial, and diagonal), and material (1.05 g/cm^3 (polystyrene), 1.27 g/cm^3 (ultem), 1.41 (delrin), and 1.56 g/cm^3 (torlon)).

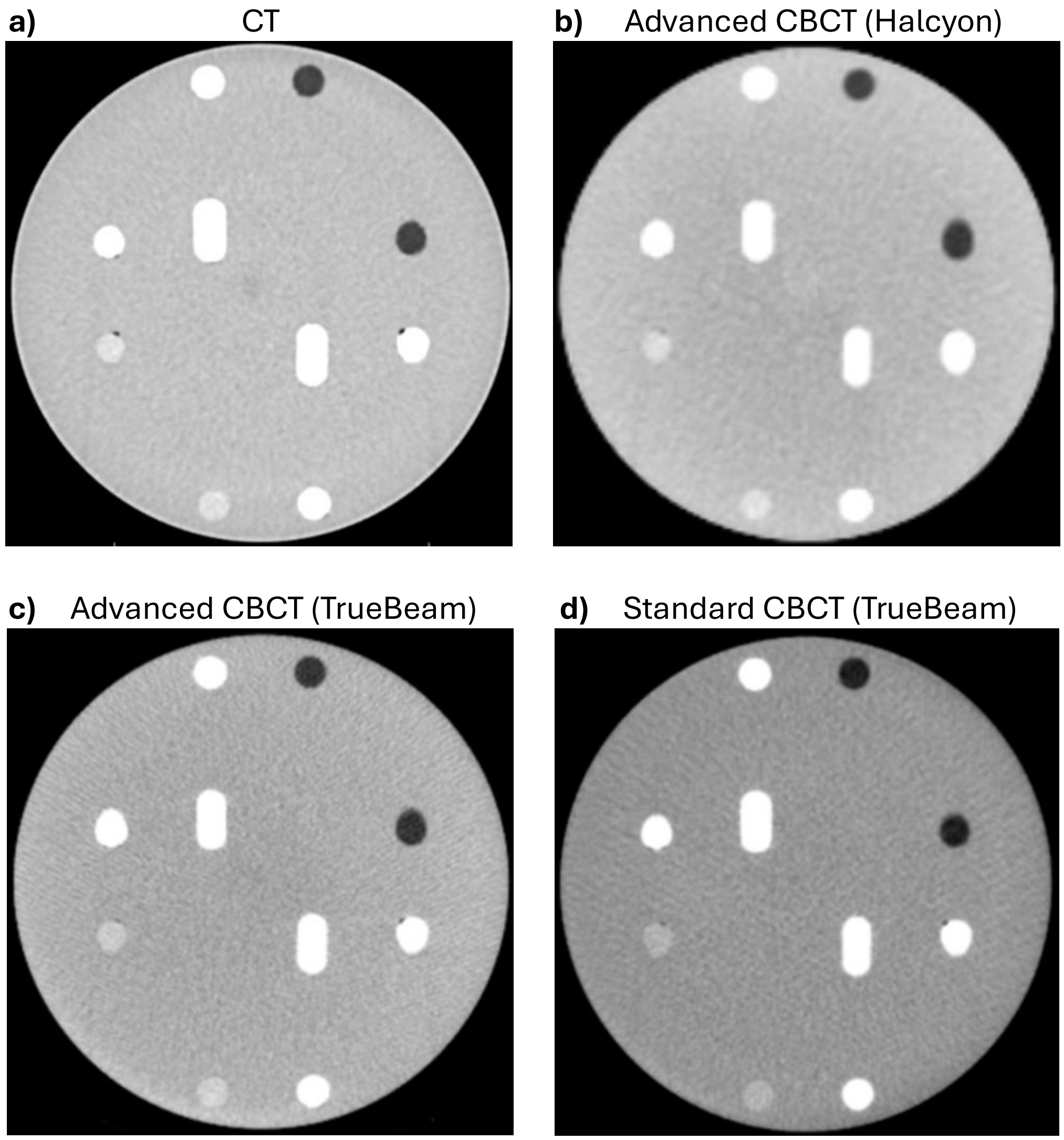

Fig. S2. Two-dimensional slices from the contrast enhancement CT (CECT) phantom [1]. Acquired on a) CT in the volume mode reconstructed with iterative algorithm AIDR3; b) advanced CBCT (Halcyon); c) advanced CBCT (TrueBeam); and standard CBCT (TrueBeam) in the full-fan configuration, reconstructed with back-projected FDK for CBCT. Imaged at 120 kV for CT and 125 kV for CBCT. Images captured at a window level of 320/50 HU using the Weasis software.

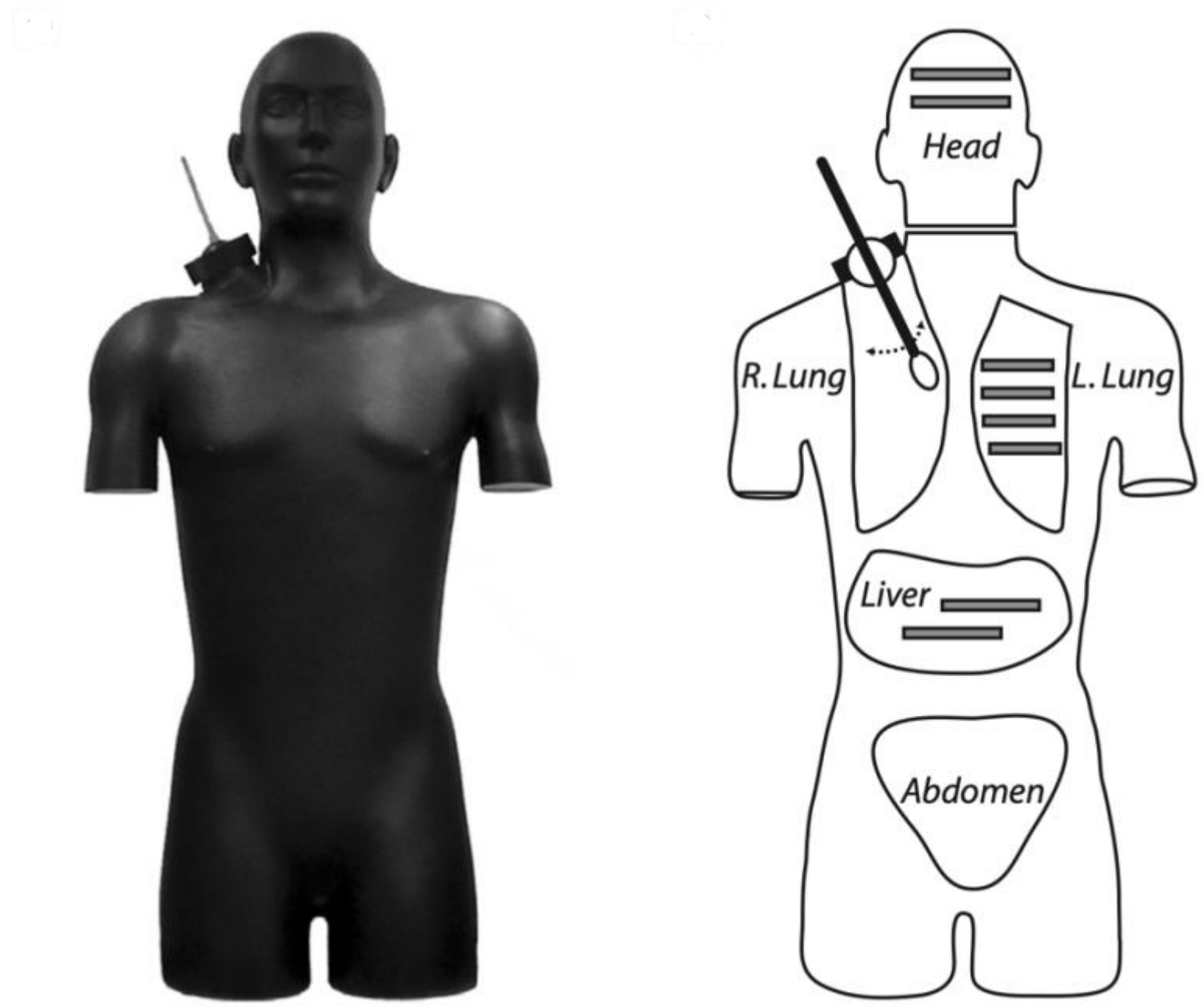

Fig. S3. Humanoid male phantom: an in-house designed anatomically accurate model of the human torso, containing major organs including the lungs and liver [2]. Corresponding to a man of total height of 1.75 m and weight of 73.5 kg. Arms and legs were excluded beyond the mid-humerus and mid-femur, respectively. The phantom was constructed from Rando® material (with CT numbers of 20–30 HU at 120 kV), and the liver constructed from Rando® polyurethane mixture. The liver contains 24 spheres mimicking physiologically relevant levels of iodine contrast enhancement and contrast-noise-ratios (CNR) in metastases with varying diameter (1.6, 3.2, 4.8, 6.4, 7.9, 9.5, and 12.7 mm) and material (acrylic or custom polyurethane with antimony to produce CT numbers of 37–131 HU at 120 kV), placed in the inferior and superior of the liver. Chiarot et al. describe further details on the phantom's materials [2].

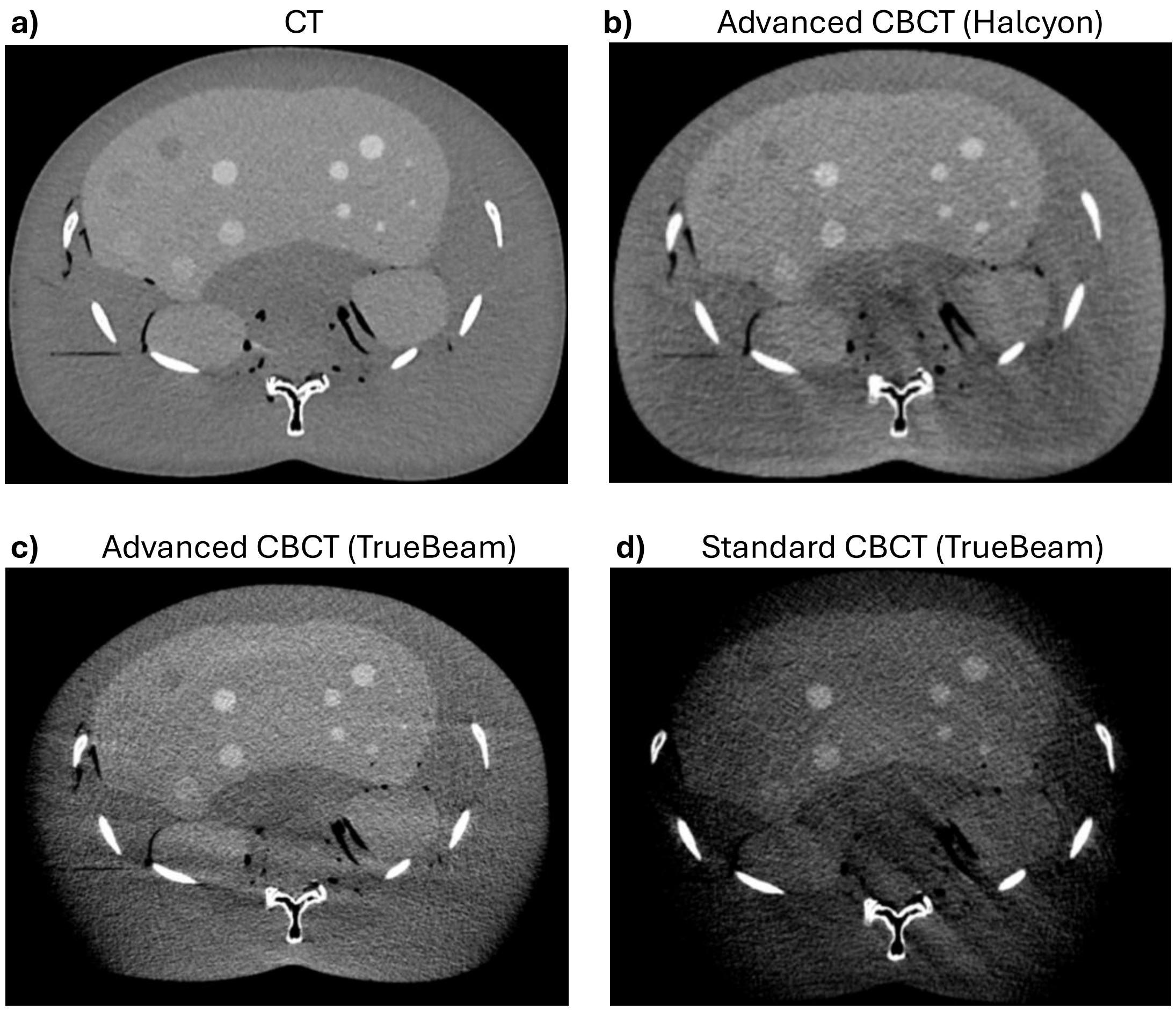

Fig. S4. Two-dimensional slices from the humanoid male phantom abdomen [2]. Acquired on a) CT in the volume mode reconstructed with iterative algorithm AIDR3; b) advanced CBCT (Halcyon); c) advanced CBCT (TrueBeam); and standard CBCT (TrueBeam) in the full-fan configuration, reconstructed with back-projected FDK for CBCT. Imaged at 120 kV for CT and 125 kV for CBCT. Images captured at a window level of 320/50 HU using the Weasis software.

Supplementary material C. MTF calculations

The directional surface-spread functions (SSFs) were assembled using voxels located within two sphere radii of the centroid. In contrast, Thornton et al. used voxels located more than eight voxel dimensions and less than three sphere radii from the centroid [3]. For each included voxel, the distance from the centroid was calculated and the distances were binned using a bin width of 1/30 of the in-plane voxel size, compared with 1/10 of the in-plane voxel size in Thornton et al. [3]. Thus, the smaller bin width provided three times as many sampling points along the SSF and a more finely sampled estimate of the surface profile. These adaptations were made because the capsules in the contrast enhancement CT (CECT) phantom were substantially smaller than the sphere in the phantom imaged by Thornton et al. [3].

Table S2. 10 % MTF threshold values for the 1.56-g/cm^3, 10-mm capsules from the contrast enhancement CT (CECT) phantom across the three capsule orientations (diagonal, axial, and transaxial). A range is reported to include values from all MTF directions (X, Y, and Z), for both replicates.

Imaging modality	Scan mode / configuration (reconstruction algorithm)	Capsule orientation	10 % MTF (lp/cm)
CT	Volume	Diagonal	3.2–4.6
		Axial	4.5–5.0
		Transaxial	4.4–5.7
	Helical	Diagonal	2.8–4.6
		Axial	3.4–6.0
		Transaxial	3.8–4.6
Advanced CBCT (Halcyon)	Half-fan (FDK)	Diagonal	2.7–3.0
		Axial	2.3–4.8
		Transaxial	2.7–3.9
	Full-fan (FDK)	Diagonal	2.6–3.3
		Axial	2.3–4.9
		Transaxial	2.7–3.8
	Full-fan (iCBCT)	Diagonal	2.8–3.7
		Axial	2.3–4.6
		Transaxial	3.6–3.9
	Full-fan (iCBCT Acuros)	Diagonal	2.8–3.7
		Axial	2.3–5.0
		Transaxial	3.5–3.9
	Full-fan (iCBCT MAR)	Diagonal	2.8–3.7
		Axial	2.3–4.9
		Transaxial	3.6–3.9
Advanced CBCT (TrueBeam)	Half-fan (FDK)	Diagonal	2.8–3.6
		Axial	2.3–4.8
		Transaxial	3.5–3.8
	Full-fan (FDK)	Diagonal	2.8–4.6
		Axial	2.3–4.7
		Transaxial	3.8–4.3
Standard CBCT (TrueBeam)	Half-fan (FDK)	Diagonal	1.8–3.6
		Axial	2.3–4.0
		Transaxial	3.4–4.5
	Full-fan (FDK)	Diagonal	2.5–3.9
		Axial	2.3–5.7
		Transaxial	3.7–4.0

Table S3. 10 % MTF threshold values for the 1.56-g/cm^3, 10-mm capsules from the contrast enhancement CT (CECT) phantom across the three different MTF directions (X, Y, and Z). A range is reported to include values from all capsule orientations (diagonal, axial, and transaxial), for both replicates.

Imaging modality	Scan mode / configuration (reconstruction algorithm)	MTF direction	10 % MTF (lp/cm)
CT	Volume	X	4.4–4.6
		Y	3.3–5.0
		Z	3.2–5.7
	Helical	X	4.3–4.6
		Y	3.4–6.0
		Z	2.8–3.8
Advanced CBCT (Halcyon)	Half-fan (FDK)	X	2.7–3.4
		Y	2.8–4.8
		Z	2.3–3.9
	Full-fan (FDK)	X	2.7–3.3
		Y	2.6–4.9
		Z	2.3–3.8
	Full-fan (iCBCT)	X	3.6–3.8
		Y	2.8–4.6
		Z	2.3–3.9
	Full-fan (iCBCT Acuros)	X	3.5–3.8
		Y	3.1–5.0
		Z	2.3–3.9
	Full-fan (iCBCT MAR)	X	3.6–3.7
		Y	2.9–4.9
		Z	2.3–3.9
Advanced CBCT (TrueBeam)	Half-fan (FDK)	X	3.6–3.7
		Y	3.0–4.8
		Z	2.3–3.8
	Full-fan (FDK)	X	4.1–4.6
		Y	3.6–4.7
		Z	2.3–3.8
Standard CBCT (TrueBeam)	Half-fan (FDK)	X	3.4–3.6
		Y	2.5–4.0
		Z	1.8–4.5
	Full-fan (FDK)	X	3.7–3.9
		Y	3.1–5.7
		Z	2.3–4.0

Supplementary material D. CT number calibration

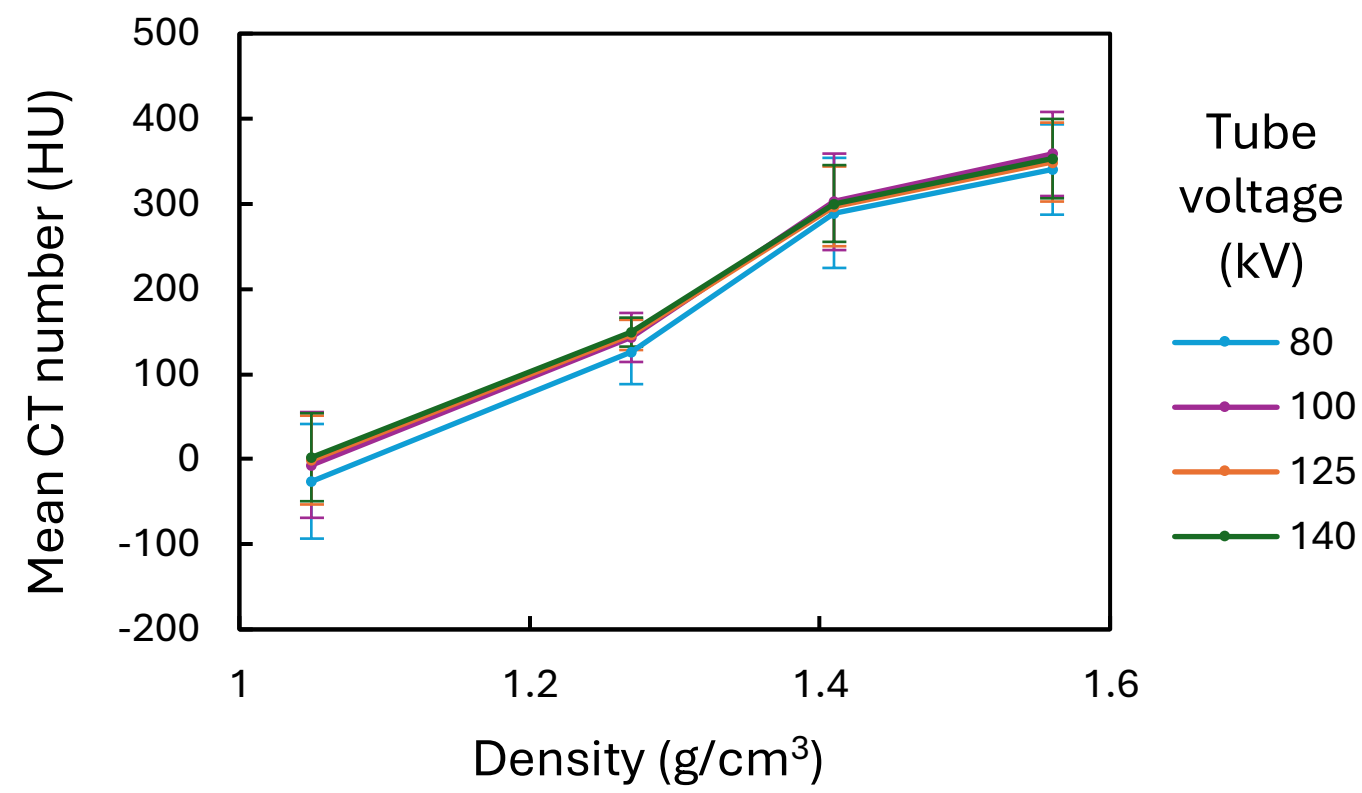

Fig. S5. Variation in mean CT number as a function of density for the axial, 10-mm capsules from the contrast enhancement CT (CECT) phantom on advanced CBCT (Halcyon) for different tube voltages. Reconstructed with back-projected FDK, and imaged in the half-fan configuration for 80–100 kV, and the full-fan configuration for 125–140 kV. Error bars represent within-capsule standard deviation of voxel-wise CT number.

Table S4. Routine quality assurance tests related to CT number calibration for each imaging modality.

Imaging modality	Clinical use	Routine quality assurance tests	Guideline/procedure
CT	Dose calculation for treatment planning	CT number accuracy (± 5 HU): daily for water (120 kV), monthly for other materials (120 kV), annually (80–135 kV)	AAPM TG-66 [4] / institutional procedures
		CT number uniformity (± 5–10 HU): daily (120 kV), monthly (80–135 kV)	
		CT number–density conversion: annually (80–135 kV)	
Advanced CBCT (Halcyon)	Dose calculation for adaptive radiotherapy	CT number constancy (± 5–8 HU): daily (most commonly used kV)	AAPM TG-179 [5] / Varian procedures / institutional procedures
		Dose-of-the-day: daily (most commonly used kV)	
		CT number accuracy (± 30–50 HU): monthly (most commonly used kV)	

		CT number uniformity (± 15 HU): monthly (most commonly used kV)	
		CT number–density conversion: regularly scheduled intervals (80–140 kV)	
Advanced CBCT (TrueBeam)	Image guidance during radiotherapy	Dose-of-the-day: daily (most commonly used kV)	AAPM TG-179 [5] / institutional procedures
		CT number uniformity (± 15 HU): monthly (most commonly used kV)	
Standard CBCT (TrueBeam)	Image guidance during radiotherapy	Dose-of-the-day: daily (most commonly used kV)	AAPM TG-179 [5] / institutional procedures
		CT number uniformity (± 15 HU): monthly (most commonly used kV)	

References

[1] Coolens C, Mohseni H, Dhodi S, Ma S, Keller H, Jaffray DA. Quantification accuracy for dynamic contrast enhanced (DCE) CT imaging: phantom and quality assurance framework. Eur J Radiol 2018;106:192-8. https://doi.org/10.1016/j.ejrad.2018.08.003.

[2] Chiarot CB, Siewerdsen JH, Haycocks T, Moseley DJ, Jaffray DA. An innovative phantom for quantitative and qualitative investigation of advanced x-ray imaging technologies. Phys Med Biol 2005;50:287-97. https://doi.org/10.1088/0031-9155/50/21/N01.

[3] Otsu N. A Threshold Selection Method from Gray-Level Histograms. IEEE Trans Syst Man Cybern 1979;9:62-6. https://doi.org/10.1109/tsmc.1979.4310076.

[4] Mutic S, Palta JR, Butker EK, Das IJ, Huq MS, Loo LN, et al. Quality assurance for computed-tomography simulators and the computed-tomography-simulation process: report of the AAPM Radiation Therapy Committee Task Group No. 66. Med Phys;30:2762-92. https://doi.org/10.1118/1.1609271.

[5] Bissonnette JP, Balter PA, Dong L, Langen KM, Lovelock DM, Miften M, et al. Quality assurance for image-guided radiation therapy utilizing CT-based technologies: a report of the AAPM TG-179. Med Phys;39:1946-63. https://doi.org/10.1118/1.3690466.
